# Clinical Characteristics and Indicators of Spontaneous Hemopneumothorax Comparing With Primary Spontaneous Pneumothorax

**DOI:** 10.7759/cureus.90423

**Published:** 2025-08-18

**Authors:** Mikito Suzuki, Kaoru Kaseda, Kaito Yano, Seiji Omura, Yu Okubo, Kyohei Masai, Tomoyuki Hishida, Keisuke Asakura

**Affiliations:** 1 Division of Thoracic Surgery, Department of Surgery, Keio University School of Medicine, Tokyo, JPN; 2 Division of Thoracic Surgery, Tokyo Metropolitan Cancer and Infectious Diseases Center Komagome Hospital, Tokyo, JPN

**Keywords:** chest x-ray, clinical indicator, primary spontaneous pneumothorax, spontaneous hemopneumothorax, video-assisted thoracic surgery

## Abstract

Introduction: This study aimed to investigate the clinical characteristics and indicators of spontaneous hemopneumothorax (SHP) compared with primary spontaneous pneumothorax (PSP).

Methods: We retrospectively evaluated patients who underwent surgery for spontaneous pneumothorax (SP) between April 2003 and April 2019. Clinical characteristics and perioperative outcomes were compared between SHP and PSP.

Results: We identified 17 (8%) and 194 (92%) patients who underwent surgery for SHP and PSP, respectively. The SHP group was significantly older (mean±standard deviation: 31±10 vs. 25±8 years; p=0.002), with a higher proportion of patients aged >30 years compared to the PSP group (53% vs. 21%; p=0.006). Furthermore, the SHP group had definitive characteristics, left-side pneumothorax (82% vs. 54%; p=0.022), smoking habits (59% vs. 29%; p=0.009), and fewer patients with episodes of ipsilateral SP (12% vs. 50%; p=0.002) than the PSP group. In SHP, the most frequent bleeding point was the superior thoracic aperture in 15 patients (88%), followed by the left mediastinum in one patient. Seven patients (41%) of the SHP group had hemodynamic instabilities and needed perioperative blood transfusion.

Conclusion: SHP had distinct clinical characteristics compared to PSP. Older age (>30 years), left-side laterality, smoking status, and a first episode of pneumothorax were feasible indicators of SHP.

## Introduction

Spontaneous hemopneumothorax (SHP) is generally deﬁned as spontaneous pneumothorax (SP) with a blood accumulation of more than 400 mL in the thoracic cavity in the absence of trauma or other obvious causes [1]. SHP often occurs in patients 20-30 years of age, often causes hemorrhagic shock, and can be life-threatening [1-10]; therefore, immediate surgical intervention is often required. A torn pleural adhesion or aberrant vessel bands between the parietal and visceral pleura develop into an SHP when the lung collapses. The prevalence of SHP often ranges from 0.5% to 6.4% among SP cases [3,11]. Owing to their uncommon nature, the clinical characteristics of SHP remain poorly understood. Furthermore, in the early course of SHP, the clinical presentation is similar to that of primary SP (PSP) [2]; therefore, recognizing the difference between SHP and PSP is important for early diagnosis. However, few reports have compared the differences in clinical characteristics between SHP and PSP [2,4]. This study compares SHP and PSP to find indicators that can help with early diagnosis and treatment.

## Materials and methods

Patient selection

**Figure 1:**
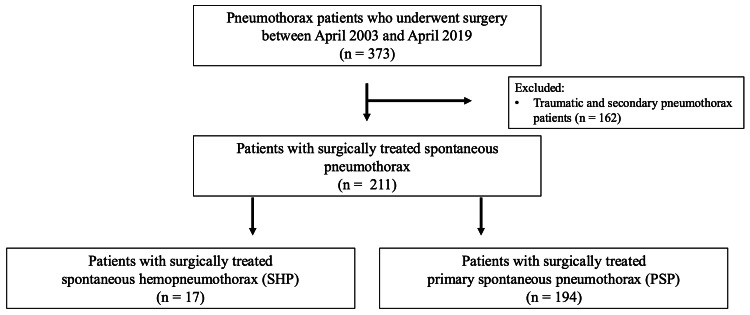
Study enrollment Two hundred and eleven patients who were surgically treated for spontaneous pneumothorax were enrolled. The spontaneous hemopneumothorax (SHP) and primary spontaneous pneumothorax (PSP) groups included 17 and 194 patients, respectively.

We retrospectively examined the records of 373 patients who underwent surgery for pneumothorax at our institute between April 2003 and April 2019. We excluded traumatic and secondary pneumothorax during the same period (Figure 1).

According to the presence or absence of hemothorax, these patients were classified into SHP and PSP groups. We defined SHP as SP with an accumulation of more than 400 mL of blood in the thoracic cavity, the total amount of preoperative chest tube, and intrathoracic clot, based on previous reports [1]. Clinical characteristics and surgical outcomes were compared between the two groups.

Data, including age at surgery, sex, laterality, body mass index (BMI), smoking status, degree of lung collapse, past history of ipsilateral SP, location of bullae, presence of hemodynamic instability, surgical procedure, operation time, duration of chest tube placement, postoperative hospital stay, postoperative complications, postoperative recurrence, amount of preoperative bleeding, amount of intrathoracic clots, bleeding point, and amount of blood transfusion, were collected from the patients’ medical records. The degree of lung collapse was classified into the following three patterns based on the guidelines of the Japan Society for Pneumothorax and Cystic Lung Disease: (a) mild, with the apex of the lung at the level of the clavicle or above; (b) moderate, between mild and severe; and (c) severe, complete lung collapse [12]. The amount of preoperative bleeding was defined as the total drainage volume from the chest tube insertion during surgery. Postoperative complications were classified following Clavien-Dindo classification (version 2.0) [13].

This study was approved by the Medical Research Ethics Committee of the Keio University School of Medicine (approval number: 20200114), and the requirement for informed consent was waived owing to the retrospective nature of the study.

Diagnosis of spontaneous hemopneumothorax

We diagnosed all the SHP and PSP cases by chest X-ray. Chest computed tomography was performed to detect bullae and intrathoracic clots. All SHP patients had a chest tube inserted under local anesthesia to drain blood. If the amount of blood drainage was extensive, the chest tube was clamped to avoid further hemorrhage based on each surgeon’s decision.

Surgical procedure

All patients were administered standard general anesthesia with a single-lung ventilation using a double-lumen endotracheal tube. Patients were placed in the lateral decubitus position. We performed three-port video-assisted thoracic surgery (VATS) as our standard procedure for the SP. First, a 2.0-cm skin incision was made in the seventh intercostal space in the middle axillary line, and a surgical port was inserted. We used a thoracoscope to explore the whole thoracic cavity. Under thoracoscopic visualization, two additional 0.5- to 2.0-cm skin incisions were made in the three or four intercostal spaces in the anterior and middle axillary lines, respectively, as maneuver ports. The size of each skin incision was according to the surgeon’s preference. Bullae were resected by an endoscopic stapler. Small bullae could be ligated or cauterized by soft coagulation electrocautery. In the SHP cases, after removing the intrathoracic clots, the aberrant blood vessels were sealed and cut using an electrical scalpel or an energy device. Then, bullae were treated. The chest tube was placed from the seventh intercostal space in the middle axillary line port.

Statistical analysis

Categorical variables were expressed as numbers (percentages) and compared using Pearson’s chi-squared test or Fisher’s exact test, as appropriate. Meanwhile, continuous variables were expressed as means±standard deviation (ranges) and compared using the Mann-Whitney U test. All tests were two-sided, and statistical significance was set at p<0.05. All statistical analyses were performed using GraphPad Prism 10 (GraphPad Software, San Diego, CA).

## Results

Clinical characteristics of patients with SHP and PSP

The baseline characteristics of patients surgically treated for SHP and PSP are shown in Table 1.

**Table 1:** Patients' characteristics BMI, body mass index; PSP, primary spontaneous pneumothorax; SD, standard deviation; SHP, spontaneous hemopneumothorax; SP, spontaneous pneumothorax; VATS, video-assisted thoracic surgery

Characteristics		SHP	PSP	p-value
		N = 17, N (%)	N = 194, N (%)	
Age, years	Mean±SD	31±10	25±8	0.002
	Range	18–53	14–49	
	Over 30	9 (53)	41 (21)	0.006
Sex	Male	16 (94)	172 (89)	0.489
Laterality	Left	14 (82)	104 (54)	0.022
BMI	Mean±SD	18±5	19±2	0.143
	Range	17–23	14–34	
Smoking status	Current or former smoker	10 (59)	56 (29)	0.015
Degree of lung collapse	≥Moderate	17 (100)	161 (83)	0.064
Past history of ipsilateral SP	Present	2 (12)	97 (50)	0.002
Past surgical history of ipsilateral SP	Present	0 (0)	27 (14)	0.100
Location of bullae	Apex	17 (100)	169 (87)	0.115

The SHP and PSP groups included 17 (8%) and 194 (92%) patients, respectively. Both groups had male predominance (94% vs. 89%; p=0.489). The SHP group was significantly older than the PSP group (mean±standard deviation (SD): 31±10 vs. 25±8 years; p=0.002), and the proportion of patients aged over 30 years was significantly different between the two groups (53% vs. 21%; p=0.006). Furthermore, the SHP group had definitive characteristics, including left-side pneumothorax (82% vs. 54%; p=0.022), smoking status (59% vs. 29%; p=0.009), and fewer patients with episodes of ipsilateral SP (12% vs. 50%; p=0.002) than the PSP group. All bullae in the SHP group were located at the apex of the lung and resected using endoscopic staplers.

Perioperative characteristics

Perioperative characteristics of SHP and PSP are shown in Table 2.

**Table 2:** Perioperative characteristics NA, not applicable; SD, standard deviation; SHP, spontaneous hemopneumothorax; SP, spontaneous pneumothorax; VATS, video-assisted thoracic surgery. * Clavien-Dindo classification (version 2.0)

Characteristics		SHP	PSP	p-value
		N = 17, N (%)	N = 194, N (%)	
Hemodynamic instability	Present	7 (41)	0 (0)	<0.001
Surgical approach	VATS	14 (82)	180 (93)	0.145
Operation time, min	Mean±SD	88±21	80±35	0.253
	Range	45–123	22–243	
Duration of chest tube placement, days	Mean±SD	2±1	4±2	0.743
	Range	1–4	0–22	
Postoperative hospital stay, days	Mean±SD	5±2	4±2	0.247
	Range	2–9	1–23	
Postoperative complication	≥Grade2*	1 (6)	16 (4)	0.538
Postoperative recurrence	Present	0 (0)	34 (18)	0.059
Amount of preoperative bleeding, mL	Mean±SD	1064±657	NA	NA
	Range	13–2200		
Amount of intrathoracic clot, mL	Mean±SD	667±610	NA	NA
	Range	20–2240		
Bleeding point	Superior thoracic aperture	15 (88)	NA	NA
	Mediastinum	1 (6)		
	Not detected	1 (6)		
Amount of blood transfusion, mL	Mean±SD	362±647	NA	NA
	Range	0–2240		

**Figure 2:**
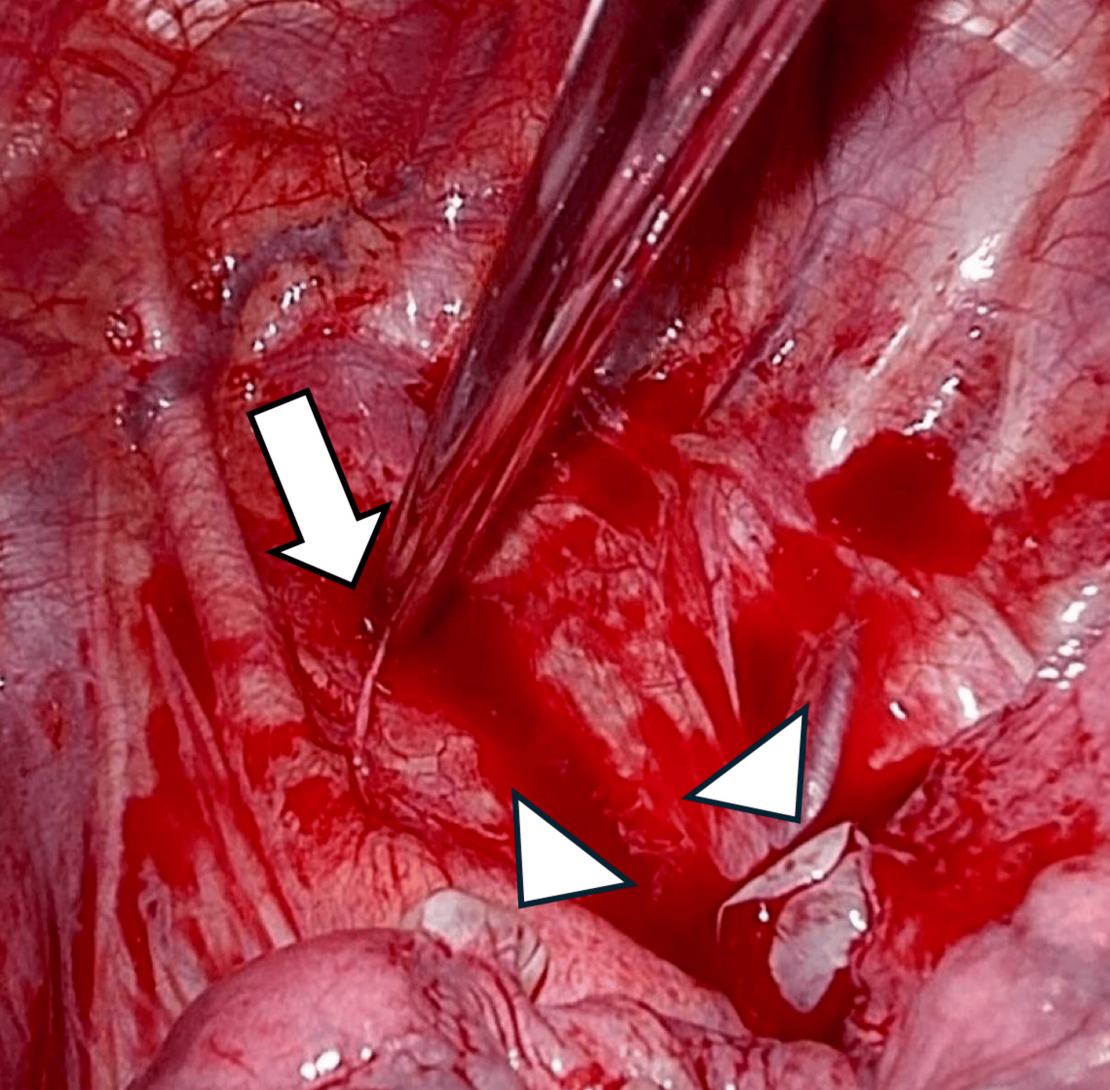
The intraoperative finding of the aberrant vessel Aberrant vessel at the superior thoracic aperture (arrow) and bullae at the apex of the lung (arrowheads).

In the SHP group, 14 patients had chest pain, two had dyspnea, and one patient had both symptoms. Furthermore, 41% of cases had hemodynamic instabilities. Eighty-two percent of SHP cases were approached by video-assisted thoracic surgery (VATS), and the others by open thoracotomy. The most frequent bleeding point was the superior thoracic aperture (15 patients, 88%), followed by the left mediastinum (one patient, 6%) and undetected sources (one patient, 6%). Fourteen patients (82%) had aberrant vessels from the chest wall that caused intrathoracic bleeding (Figure 2).

Among them, two (11%) patients had a hemorrhage from the pulmonary parenchyma attached to the torn aberrant vessels. The mean amount of preoperative bleeding was 1,064±657 (range: 13-2,200) mL, whereas the intrathoracic clot of 667±610 (range: 20-2,240) mL remained intraoperatively. After surgery, the duration of chest tube placement and postoperative stay were not significantly different between the two groups. There were no significant differences in postoperative complications or recurrence between the two groups, possibly because of the small sample size. In one case of SHP, postoperative grade 2 atrial fibrillation was observed. The time between onset to hospital arrival and hospital arrival to surgery was 11.9±19.6 (range: 0-81.5) hours and 35.9±54.2 (range: 1.0-209.0) hours, respectively.

Representative case of delayed surgical intervention for SHP

**Figure 3:**
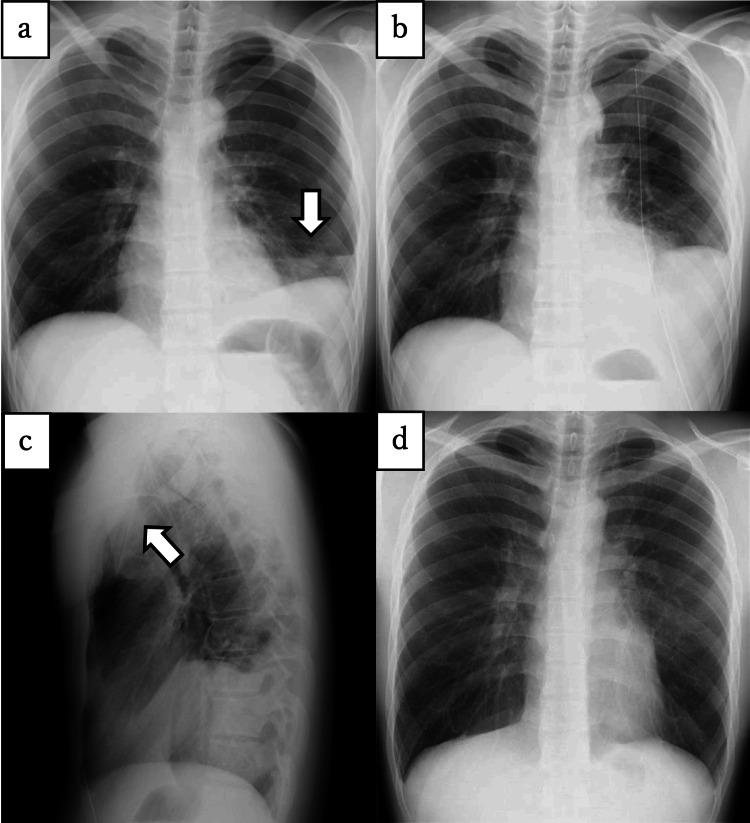
Representative case of delayed surgical intervention for spontaneous hemopneumothorax (SHP) (a) Initial chest X-ray showed moderate pneumothorax and small air-fluid level (arrow), but SHP was not suspected by primary care doctor. (b) Half a day later, after blood drainage presented. Follow-up X-ray revealed an increase of air-fluid level and the patient was diagnosed with SHP. (c) Chest tube was positioned ventrally (arrow); therefore, the hemorrhage drained belatedly. (d) Postoperative chest X-ray.

Figure 3 shows a representative case of delayed surgical intervention for SHP.

A 20-year-old non-smoking male with no medical history had left chest pain and presented to our hospital one hour later. An initial chest X-ray at 22:00 showed SP and a small air-fluid level (Figure 3a); however, SHP was not strongly suspected by the patient’s primary care doctor. Moreover, in spite of prompt chest drain insertion for SP, intrathoracic bleeding was not thoroughly drained due to the ventral chest tube position. Consequently, the patient underwent conservative treatment, and half a day later, at 12:30 the next afternoon, the blood drainage suddenly emerged, and follow-up chest X-ray revealed an increased air-fluid level (Figures 3b-3c). The patient’s blood pressure gradually decreased, and when SHP was finally diagnosed, emergent surgery was performed. During the surgery, we found an intrathoracic clot of >1,000 mL. After suctioning, we identified minor continuous arterial bleeding from the aberrant vessel at the superior thoracic aperture. A bulla was also found at the apex of the lung. The aberrant vessel was sealed, and the bulla was resected using an endoscopic stapler. The patient's postoperative course was uneventful. The chest tube was removed on postoperative day two, and the patient was discharged on postoperative day five (Figure 3d).

## Discussion

This study revealed the clinical characteristics of rare SHP and identified the indicators of SHP relative to PSP. Univariate analysis revealed that older age (>30 years), left-side pneumothorax, smoking status, and a first episode of pneumothorax were significant indicators of SHP. The proportion of SHP was 8% of the surgically treated SP cases, with SHP being predominant in young male patients, which was consistent with previous reports [1-10].

The mechanism of SHP is primarily due to the disconnection of pleural adhesions or aberrant vessel bands between the parietal and visceral pleura. We intraoperatively found the bleeding point in 94% of cases, with the superior thoracic aperture being the most common site (88%), followed by the left superior mediastinum (6%). Similar results were previously reported, with the superior thoracic aperture in 65.4% of cases and the left superior mediastinum in 23.1% of cases. In contrast, regarding the superior mediastinum, no right-sided bleeding points were present [2]. Aberrant vessels were identified in 82% cases. A previous report identified bleeding points in 75% of SHP, with 50% arising from aberrant vessels [3]. Another bleeding mechanism due to the rupture of vascularized bullae was also reported [3,4], but we did not detect any such cases. Furthermore, we found bleeding from the lung parenchyma attached to an aberrant vessel in 11% cases, consistent with previous reports of 4%-10% [2,3,5]. Thus, a cautious exploration of not only the parietal pleura and aberrant vessels but also the visceral pleura should be performed to prevent excess hemorrhage during surgery.

Due to being an uncommon condition, the clinical characteristics of SHP have not been adequately described; moreover, few studies have compared the clinical characteristics of SHP and PSP [2,3]. A previous report demonstrated that smoking status, height, and BMI were significant factors of SHP in univariate analysis [3]. High rates of smoking in patients with SHP have been previously reported in several studies [2-4]. Cigarette smoking is a well-known cause of PSP [14] and intrathoracic inflammatory reaction [15]. Among PSP patients, smoking-induced inflammation leads to intrathoracic adhesions and angiogenesis, which then develop into SHP. Moreover, PSP-induced exposure of the pleural space to environmental air and mechanical stretching of the pleura could also result in an inflammatory reaction [15]. Furthermore, the representation of an older population in the SHP group compared to that in the PSP group was a remarkable aspect of the present study. The findings herein may be influenced by higher smoking status; therefore, further case accumulation is needed.

A history of SP appears to be related to a higher risk of SHP; however, a first episode of SP was the strongest indicator of SHP in our study. A high incidence of no previous history of SP in SHP has been reported [2,3,6,8,9]. This does not indicate that SHP patients did not have a history of SP at all, but may have been asymptomatic with undiagnosed SP occurring and developing into an intrathoracic inflammatory reaction. As a result, when SHP developed, the patients were diagnosed as “first SP”. The trend of SHP toward older ages than in PSP in our univariate analysis may explain the greater chance of having experienced repeated asymptomatic SP.

Why SHP presents with left-sided predominance remains unclear. A laterality predominance of SHP was controversial [1,2,6,10,16]. Onuki et al. have suggested that the major arteries or the aortic arch are located in the left thoracic cavity and can be a source of blood supply [2]. Furthermore, we hypothesize that the anatomic structures may also be associated with a left-sided predominance of SHP. We found that the left mediastinum, especially the superior mediastinum, had a bumpier surface than the right mediastinum due to the intrathoracic structures, including the heart, aortic arch, and great vessels such as the left brachiocephalic vein, common carotid artery, and subclavian artery (Figure 3).

**Figure 4:**
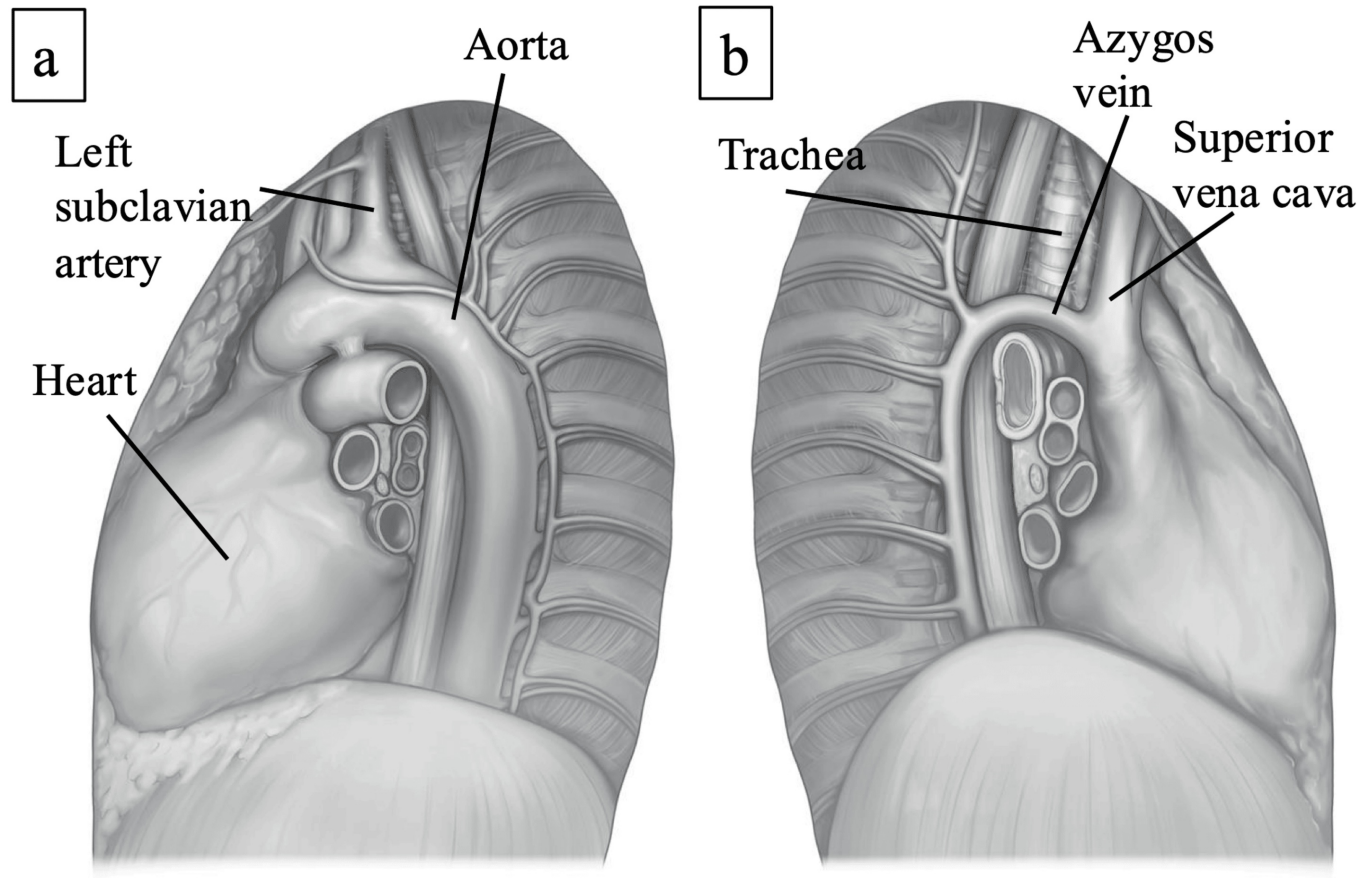
The anatomy of intrathoracic structures The surface of the left-sided thoracic cavity (a) is bumpier in comparison to the right side (b) due to the left intrathoracic structures, including the heart, aortic arch, and great vessels (both figures are original images).

When asymptomatic SP improves, the lung parenchyma physically rubs against the bumpy superior mediastinal surface, where irregular adhesions may be promoted. The beating of the great arteries may also relate to rubbing against the lung parenchyma. This also explains the above-mentioned SHP prevalence in the left superior mediastinum but not in the right.

There are currently no guidelines for the treatment of SHP. Whether prompt surgery or conservative therapy is better remains controversial [3,7-9,17-19]. The surgical indications for SHP are as follows: continuous bleeding (>100-150 mL/h), a large amount of residual intrathoracic clots, and signs of hypovolemic shock [1]. A conservative approach was attempted for patients with no residual intrathoracic hematoma or a good lung expansion. However, conservative management is associated with increased rates of blood transfusion, failure of lung re-expansion, and hematoma infections [3,7-9,16]. A longer duration of tube drainage and hospital stay was also reported [16]. SHP caused severe hemodynamic instability, as shown in 15%-47% of SHP patients required blood transfusion; however, the postoperative outcomes were not significantly worse than those of SP using appropriate surgical treatment. We support that prompt surgery for SHP could prevent extra bleeding and blood transfusion and obtain better outcomes [1-3,5-10,16]. VATS was a reliable procedure for SHP, as well as the less invasive uniportal VATS, which supports early surgical intervention [19].

Early diagnosis of SHP is sometimes challenging due to its radiological similarity with PSP. The air-fluid level was pivotal in the diagnosis of SHP on chest X-ray [4]; however, SP was the only radiological finding in 10% of SHP cases [20]. Therefore, to avoid overlooking SHP, it is essential to comprehend not only the radiological features but also the clinical characteristics. Although the patient presented in Figure 2 should have been diagnosed with SHP in the initial chest X-ray, recognizing the relevant risk factors, including the left-side pneumothorax and no past history of SP, could help to take a close repeated radiological follow-up and prevent the delay of surgery. Moreover, in the emergency room, the initial treatment of SP is not performed by only thoracic surgeons; hence, all the physicians involved with SP broadly need to share an understanding of this rare entity. Moreover, we may encourage hospitalization for patients with our indicative factors to check the near future bleeding.

This study has a few limitations. First, it was a retrospective, single-center study design. Second, the sample size was small (although the sample size of 17 patients with SHP in this study was similar to previous reports); hence, only univariate analysis was conducted for identifying the clinical indicators of SHP. Third, we excluded secondary pneumothorax in this study because SHP were predominant in the younger population. However, not all SHP cases included secondary pneumothorax in our study; therefore, the detailed etiology of SHP still remains unclear, and further case accumulation was needed.

## Conclusions

This study identified the clinical characteristics and indicative factors of SHP by comparing it to PSP. Older age (especially over 30 years), left-sided pneumothorax, smoking habits, and first episode of pneumothorax were identified as risk factors of SHP. SHP is a rare but potentially life-threatening entity; however, early diagnosis of SHP is sometimes challenging due to its radiological similarity with PSP. Moreover, the initial treatment of SHP is not performed only by thoracic surgeons but also by physicians and emergency physicians. Recognizing clinical indicators may facilitate the appropriate preoperative evaluation of SHP for even non-specialists of SP.
